# Circular American Medical Association

**Published:** 1851-09

**Authors:** 


CIRCULAR.

We invite attention to the following circular.

Sir:—The committee of publication respectfully submit to you as a member of the American Medical Association, the following statement: The whole amount received from the assessment for 1851 has been about six hundred dollars, which, with the balance in the treasury at the last report (four hundred dollars,) makes one thousand dollars.

It is estimated that the cost of Vol. IV. will be about seventeen hundred dollars; the expense of printing the Reports being one thousand dollars, and of the Prize Essay, with the necessary illustrations, nearly seven hundred dollars.

But the committee are without the means for that purpose, and they appeal to the Association to furnish them. A large number of copies of the three volumes already published remain unsold, and, if the members will complete their sets, and use their influence to extend the sale of these volumes, the reqaired sum may be readily raised.

The Committee call the attention of the Association to the terms upon which the published volumes are now furnished to members, or to societies which have been represented in the Association.

| | |
|---|---|
| Either of the first three volumes separately (paper covers) | $1 50 |
| A complete set in three valumes (paper covers) - - | 4 00 |
| Do. do do. (cloth) - - - . | 5 00 |
| Single copies of vol. iv. (to permanent members) - | 2 00 |
| Three do. do. do. - - - - - | 5 00 |

In all cases, the amount must be remitted to the Treasurer of the Association, Isaac Hays, M.D., Philadelphia.

Your earliest possible attention to the above is earnestly solicited, to avoid great delay in the publication of the forthcoming volume of the Transactions.

ISAAC HAYS, M. D.,
**Ch'm of the Com. of Pub. Am. Med. Ass., and Treasurer.**

*Philadelphia, Aug.* 1, 1851.

